# The Multi-Level Action of Fatty Acids on Adiponectin Production by Fat Cells

**DOI:** 10.1371/journal.pone.0028146

**Published:** 2011-11-29

**Authors:** Shakun Karki, Partha Chakrabarti, Guanrong Huang, Hong Wang, Stephen R. Farmer, Konstantin V. Kandror

**Affiliations:** Boston University School of Medicine, Boston, Massachusetts, United States of America; Sapienza University of Rome, Italy

## Abstract

Current epidemics of diabetes mellitus is largely caused by wide spread obesity. The best-established connection between obesity and insulin resistance is the elevated and/or dysregulated levels of circulating free fatty acids that cause and aggravate insulin resistance, type 2 diabetes, cardiovascular disease and other hazardous metabolic conditions. Here, we investigated the effect of a major dietary saturated fatty acid, palmitate, on the insulin-sensitizing adipokine adiponectin produced by cultured adipocytes. We have found that palmitate rapidly inhibits transcription of the adiponectin gene and the release of adiponectin from adipocytes. Adiponectin gene expression is controlled primarily by PPARγ and C/EBPα. Using mouse embryonic fibroblasts from C/EBPα-null mice, we have determined that the latter transcription factor may not solely mediate the inhibitory effect of palmitate on adiponectin transcription leaving PPARγ as a likely target of palmitate. In agreement with this model, palmitate increases phosphorylation of PPARγ on Ser273, and substitution of PPARγ for the unphosphorylated mutant Ser273Ala blocks the effect of palmitate on adiponectin transcription. The inhibitory effect of palmitate on adiponectin gene expression requires its intracellular metabolism *via* the acyl-CoA synthetase 1-mediated pathway. In addition, we found that palmitate stimulates degradation of intracellular adiponectin by lysosomes, and the lysosomal inhibitor, chloroquine, suppressed the effect of palmitate on adiponectin release from adipocytes. We present evidence suggesting that the intracellular sorting receptor, sortilin, plays an important role in targeting of adiponectin to lysosomes. Thus, palmitate not only decreases adiponectin expression at the level of transcription but may also stimulate lysosomal degradation of newly synthesized adiponectin.

## Introduction

Major discoveries of the last 15 years have established fat as an important secretory tissue. It is now clear that adipokines regulate central aspects of metabolism, such as body weight, energy expenditure, insulin sensitivity and a variety of physiological conditions which are directly or indirectly associated with these parameters. In particular, adiponectin that is generally considered as one of the most abundant and functionally significant adipokines improves whole body insulin sensitivity and glucose tolerance [1], [2], [3].

Unlike many other adipokines, secretion of adiponectin is decreased in obesity [1], [4], [5], [6], [7]. This observation is highly significant because it may help to understand a long-known connection between obesity, insulin resistance and diabetes, a disease that has reached pandemic proportions in the recent years [8]. In further support of this model, adiponectin has been shown to improve insulin resistance associated with obesity [9], [10]. This suggests that a decrease in adiponectin production in obese individuals may represent an important causative factor in the development and/or propagation of the insulin resistance, diabetes, and, potentially, other metabolic diseases.

How exactly excess fat suppresses production of adiponectin is not understood mechanistically. However, since obesity is usually accompanied by elevated levels of circulating fatty acids and, in particular, saturated fatty acids (SFA), such as palmitate, the negative effect of obesity on adiponectin may be attributed to the direct action of fatty acids on adipocytes [11], [12], [13]. In agreement with this hypothesis, we have found that palmitate inhibits adiponectin production by adipocytes in a cell-autonomous fashion. Interestingly, palmitate exerts its effect on fat cells at different levels. First, palmitate rapidly decreases adiponectin transcription. This effect is associated with phosphorylation of PPARγ on Ser273. In addition, palmitate may increase degradation of *de novo* synthesized adiponectin in lysosomes *via* a sortilin-mediated mechanism.

## Results

### Metabolic products of palmitate inhibit adiponectin expression at the level of transcription

**Figure 1:**
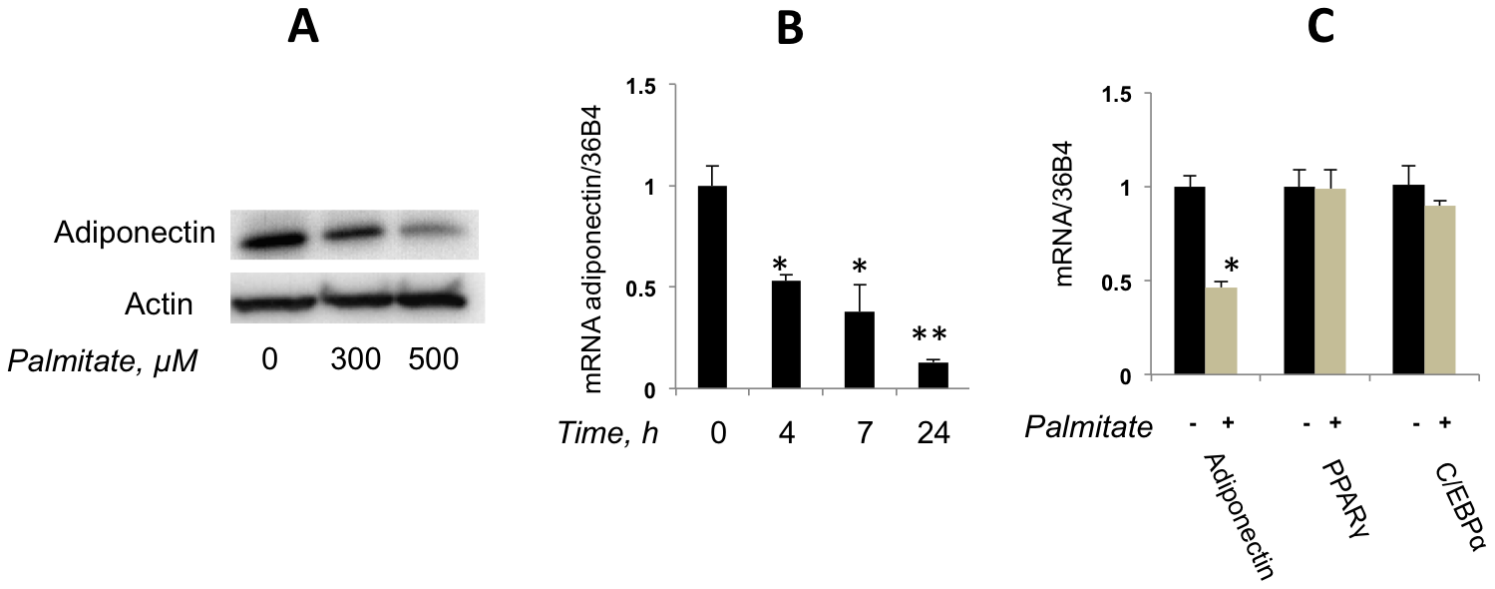
Palmitate decreases adiponectin expression and secretion. (A) Differentiated 3T3-L1 adipocytes were incubated with and without palmitate for 4 hours, and the presence of adiponectin in cell media was analyzed by Western blotting. Intracellular actin was used as a loading control. (B) Adiponectin mRNA in 3T3-L1 adipocytes incubated with and without 500 µM of palmitate for indicated periods of time. (C) Expression levels of mRNAs for adiponectin, PPARγ, and CEBPα in differentiated 3T3-L1 adipocytes incubated in the presence and in the absence of 500 µM palmitate for 4 hours. In (B) and (C), mRNA was analyzed by quantitative PCR and normalized by 36B4. Data were expressed as mean ± S.D. * p<0.05; ** p<0.001.

Fig. 1A shows that palmitate at a concentration of 300–500 µM strongly decreases secretion of adiponectin from 3T3-L1 adipocytes. Although blood levels of palmitate *in vivo* vary within 100–150 µM [14], the total concentration of free fatty acids may significantly exceed 1 mM [15], [16], so palmitate concentrations used in our study may not be far from physiological.

In order to determine whether palmitate inhibits adiponectin production at the level of gene expression, we have measured levels of adiponectin mRNA in palmitate-treated and not treated adipocytes by qPCR. As is shown in Fig. 1B, palmitate decreases adiponectin mRNA in a time-dependent fashion. In all following experiments, we have incubated adipocytes with palmitate for no longer than 4 h, because at this time point palmitate does not affect mRNAs for PPARγ and C/EBPα (Fig. 1C) – the two major transcription factors that control expression of the adiponectin gene [17]. Although we determined that oleate also inhibited adiponectin expression and secretion (Fig. S1), in this study, we decided to focus on the effect of a common dietary saturated fatty acid, palmitate.

**Figure 2:**
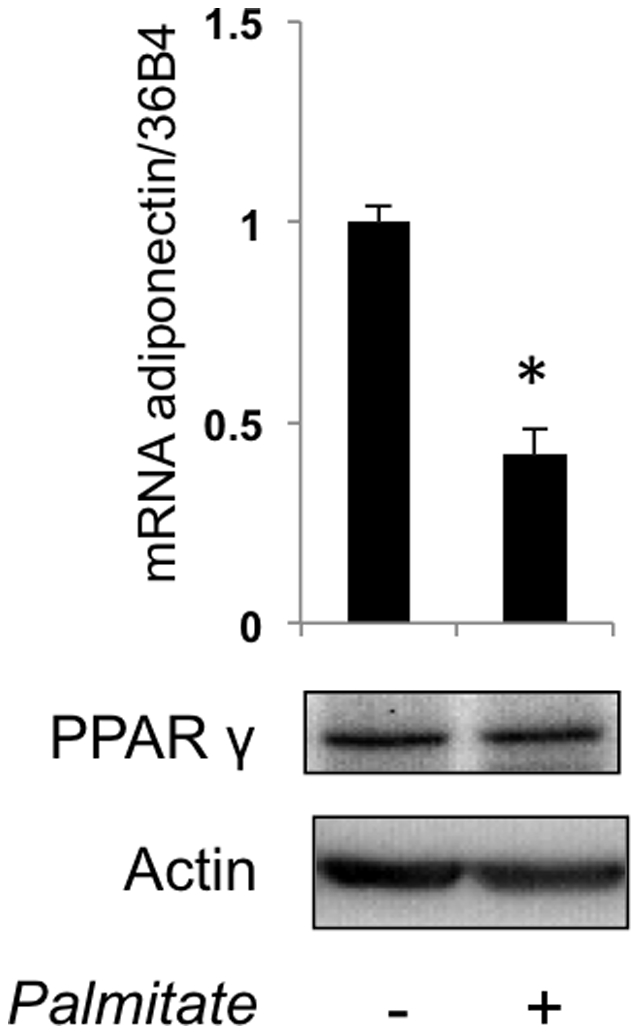
Palmitate decreases adiponectin mRNA in C/EBPα null mouse embryonic fibroblasts. Top panel: Differentiated MEFs from C/EBPα null mice were incubated with and without 500 µM palmitate for 4 h, and adiponectin mRNA was analyzed by quantitative PCR and normalized by 36B4. Data were expressed as mean ± S. D. * p<0.05. Bottom panels: cell lysate was prepared from C/EBPα null MEFs and analyzed by Western blotting (35 µg of total protein per lane).

The adiponectin promoter has several C/EBPα enhancer elements [17]. To determine if the effect of palmitate on adiponectin gene expression is mediated by C/EBPα, we used differentiated mouse embryonic fibroblasts obtained from C/EBPα–null mice [18]. Note, that these cells express detectable levels of PPARγ (Fig. 2). Although total adiponectin expression in these cells was decreased (not shown), we found that palmitate was still able to suppress expression of adiponectin mRNA (Fig. 2), suggesting that C/EBPα may not be solely responsible for this effect. Therefore, we decided to test an alternative hypothesis that palmitate decreases expression of adiponectin mRNA *via* PPARγ.

**Figure 3:**
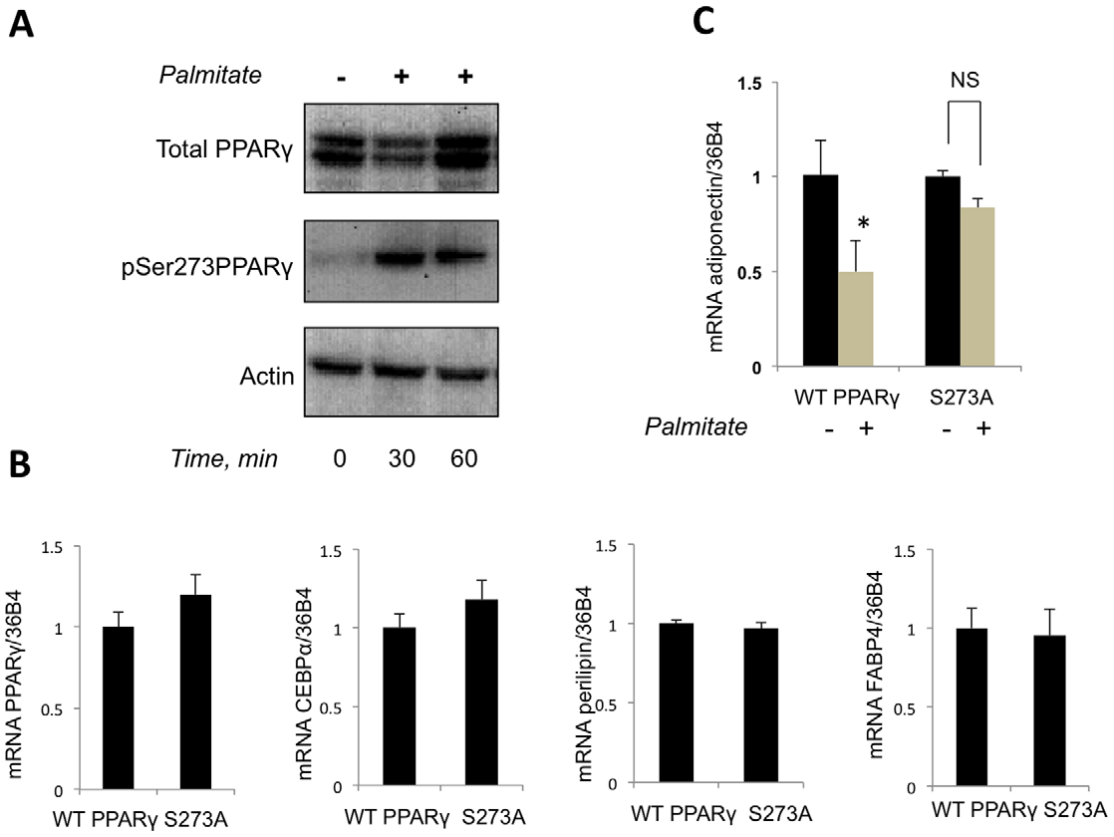
Palmitate inhibits adiponectin transcription by increasing phosphorylation of PPARγ on serine 273. (A) Differentiated 3T3 L1 adipocytes were serum starved for 2 hours and treated or not treated with palmitate for indicated periods of time. Cell lysates were analyzed by Western blotting (35 µg of total protein per lane). (B) Swiss fibroblasts stably transfected with wild-type (WT) and S273A PPARγ were differentiated as described in Materials and Methods and mRNAs for PPARγ, C/EBPα, perilipin and FABP4 were determined by quantitative PCR and normalized by 36B4 levels. (C) Differentiated Swiss fibroblasts stably transfected with wild-type (WT) and S273A PPARγ were incubated with 500 µM of palmitate for 5 hours. Adiponectin mRNA was determined by quantitative PCR and normalized by 36B4 levels. Data were expressed as mean ± S. D. * p<0.05.

In agreement with several previously published reports summarized in [17], we found that over-expression of PPARγ increases, while knock down of PPARγ – decreases adiponectin mRNA (not shown) which confirmed the involvement of PPARγ in regulation of the adiponectin gene expression. A recent study from the Spiegelman's laboratory, suggested that adiponectin transcription may be regulated by Cdk5-mediated phosphorylation of PPARγ on Ser273 [19]. In agreement with this report, we have found that palmitate stimulates phosphorylation of PPARγ on Ser273 (Fig. 3A). To determine whether this phosphorylation event is sufficient to convey the negative effect of palmitate on adiponectin transcription, we used differentiated Swiss fibroblasts stably transfected with wild type PPARγ or unphosphorylatable mutant of PPARγ where Ser273 was substituted for Ala. As is shown in Fig. 3B, the extent of differentiation of both cell lines was identical as judged by the expression levels of several differentiation markers: PPARγ, C/EBPα, perilipin, and FABP4. Interestingly, palmitate decreased adiponectin transcription in the former cell line but not in the latter (Fig. 3C).

Thus, phosphorylation of PPARγ on Ser273, presumably by Cdk5 [19], may be responsible for the effect of palmitate on the adiponectin gene expression. However, the upstream mechanism of palmitate action remains unknown. According to one model, palmitate may activate toll-like receptor 4 in adipocytes [20], [21] and trigger a signal transduction pathway that leads to phosphorylation of PPARγ on Ser273. On the other hand, palmitate may penetrate inside the cell and become converted into lipid products, such as palmityl-CoA, diacylglycerol or ceramide. These compounds induce multiple cellular responses, such as activation of protein kinases C and IκB, stimulation of ROS production, ER stress, etc. that are collectively known as lipotoxicity [22], [23], [24], [25].

The first step of FA metabolism is the conversion into acyl-CoA by the family of enzymes called acyl-CoA synthetases [26]. In particular, palmitate is the preferential substrate for acyl-CoA synthetase 1, or ACSL1, the major acyl-CoA synthetase in adipocytes [26]. Thus, in order to determine whether the effect of palmitate on adiponectin gene expression represents the result of the activation of the cell surface receptors or is caused by intracellular lipotoxicity, we used the stable line of 3T3-L1 adipocytes where expression of ACSL1 was significantly attenuated by constitutive production of a ACSL1-shRNA [27]. Note, that FA uptake into these cells is not altered, while palmitate-CoA synthetase activity is dramatically decreased [27].

**Figure 4:**
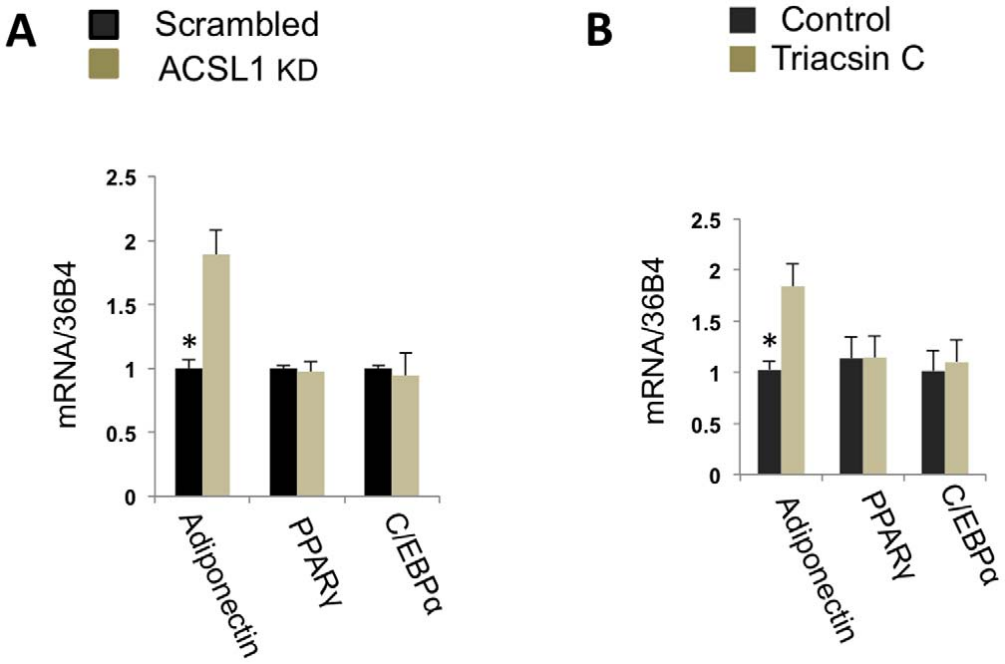
Long-chain acyl CoA synthetase negatively regulates adiponectin mRNA in adipocytes. (A) Long-chain acyl CoA synthetase was stably knocked down in 3T3-L1 adipocytes with the help of shRNA (ACSL1 KD), and mRNAs for adiponectin, PPARγ, and CEBPα were analyzed by quantitative PCR in ACSL1 KD and control (scrambled shRNA) cells. (B) Differentiated wild type 3T3-L1 adipocytes were incubated with and without 48 µM of Triacsin C for 6 hours, and mRNAs for PPARγ, CEBPα and adiponectin were analyzed by quantitative PCR. Data are expressed as mean ± S. D. *p<0.05.

**Figure 5:**
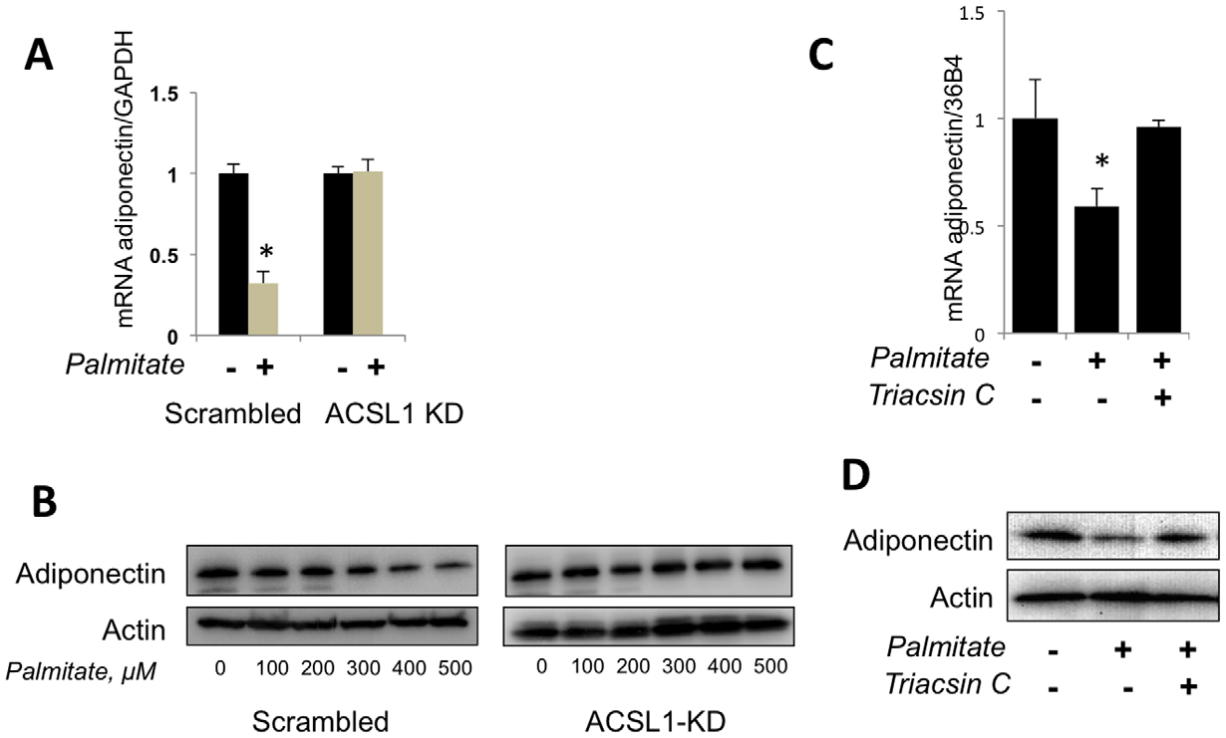
Long-chain acyl CoA synthetase mediates the inhibitory effect of palmitate on transcription and secretion of adiponectin. (A) ACSL1 KD and control (scrambled) adipocytes were incubated in the presence or absence of 500 µM palmitate for 4 h, and adiponectin mRNA was analyzed by quantitative PCR and normalized by GAPDH. Data are expressed as mean ± S. D. *p<0.05. (B) ACSL1 KD and control (scrambled) adipocytes were incubated with indicated concentrations of palmitate for 4 h, and the presence of adiponectin in cell media was analyzed by Western blotting. Intracellular actin was used as a loading control. (C) Wild type 3T3-L1 adipocytes were incubated with and without 500 µM palmitate and 48 µM Triacsin C for 4 h, and adiponectin mRNA was analyzed by quantitative PCR and normalized by 36B4. Data are expressed as mean ± S. D. *p<0.05. (D) Wild type 3T3-L1 adipocytes were incubated with and without 500 µM palmitate and 48 µM Triacsin C for 4 h, and the presence of adiponectin in cell media was analyzed by Western blotting. Intracellular actin was used as a loading control.

**Figure 6:**
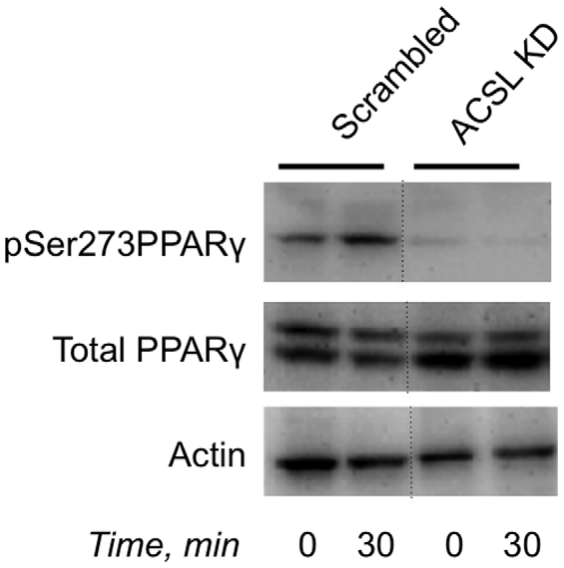
Phosphorylation of PPAR γ on serine 273 is compromised in ACSL1 KD adipocytes. ACSL1 KD and control (scrambled) adipocytes were serum starved for 2 h, treated or not treated with 500 µM palmitate for 30 min and analyzed by Western blotting (35 µg of total protein per lane). Dotted lines indicate that irrelevant lanes have been spliced out.

We have found that stable knock down of ACSL1 does not change the levels of mRNAs for PPARγ and C/EBPα but increases transcription of adiponectin (Fig. 4A). In order to confirm this observation using an independent approach, we used the acyl-CoA synthetase inhibitor, Triacsin C, and obtained a similar result (Fig. 4B). Furthermore, we have determined that palmitate does not inhibit transcription of adiponectin mRNA and secretion of adiponectin from ACSL1-depleted cells (Fig. 5A,B). Also, treatment of cells with Triacsin C prevents the inhibitory effect of palmitate on adiponectin transcription and secretion (Fig. 5C,D). Interestingly, palmitate-stimulated phosphorylation of PPARγ on Ser273 is compromised in ACSL1-depleted cells (Fig. 6). Based on these results, we suggest that inhibition of adiponectin gene expression by palmitate is caused by intracellular lipotoxicity rather than by binding of palmitate to the extracellular receptors. Such a conclusion would be consistent with a recent paper by Loo et al. [28] demonstrating a strong negative correlation between adiponectin expression and lipid accumulation in individual sub-populations of 3T3-L1 adipocytes *in vitro*.

### Palmitate promotes degradation of adiponectin via the lysosomal pathway

Inhibition of gene transcription may not be the only mechanism by which fatty acids suppress adiponectin production and secretion from adipocytes. In fact, Nguyen et al. noticed that fatty acids decrease adiponectin secretion within 1 hour of incubation [12]. Clearly, such a rapid effect of fatty acids may only be related to a very late post-translational regulatory step in adiponectin production.

**Figure 7:**
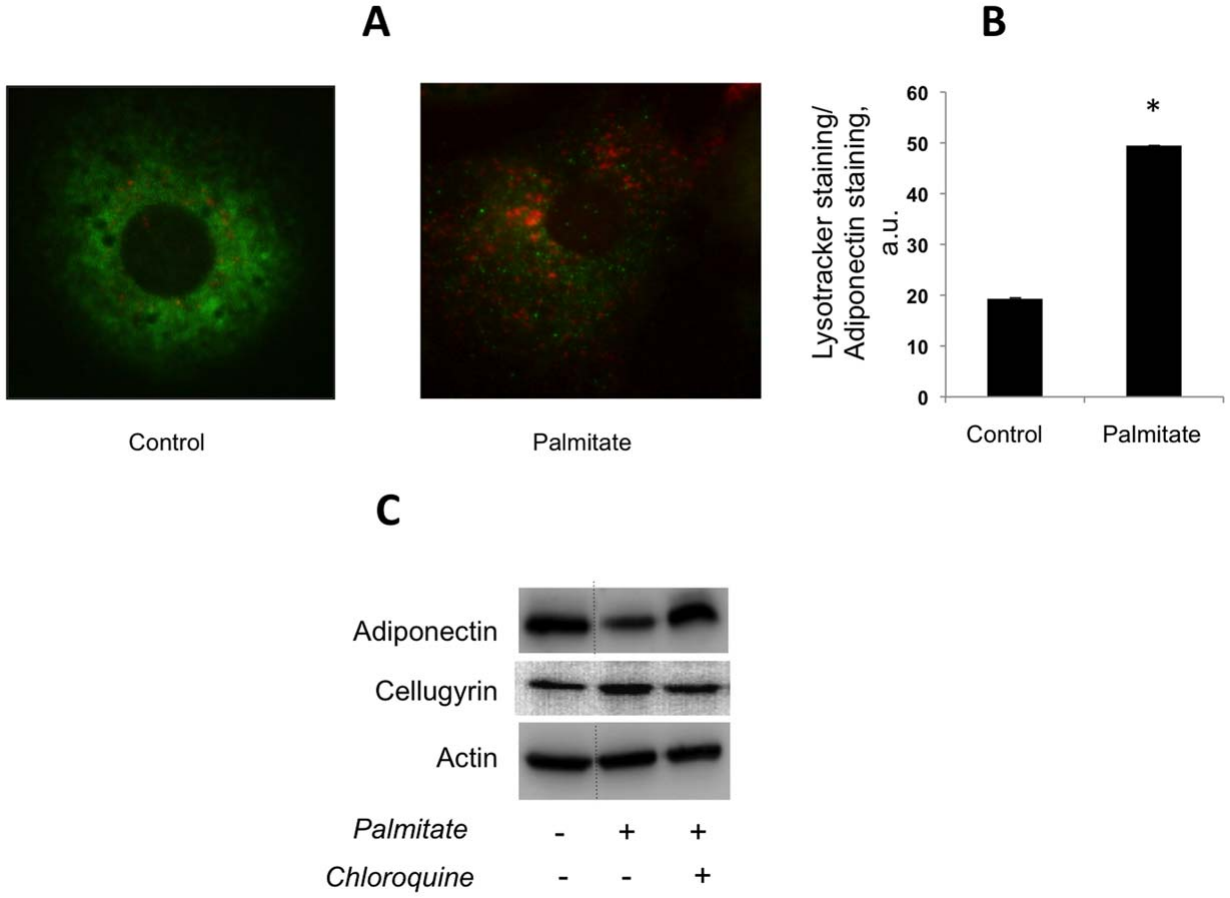
Palmitate increases intracellular degradation of adiponectin *via* the lysosomal pathway. (A) Wild type 3T3-L1 adipocytes were treated or not treated with 500 µM palmitate for 4 hours and then - with 100 nM of lysotracker (red) for 30 minutes at 37°C. Cells were fixed and stained for adiponectin (green). (B) The ratio between lysotracker (red) and adiponectin (green) staining was determined with the help of the ImageJ software in ca. 100 adipocytes treated and not treated with palmitate. (C) Wild type 3T3-L1 adipocytes were treated or not treated with 500 µM of palmitate and 20 µM of Chloroquine for 4 h as indicated, and the presence of adiponectin in cell media was analyzed by Western blotting. Intracellular actin was used as a loading control. Dotted lines indicate that irrelevant lanes have been spliced out.

As the major pool of intracellular adiponectin resides in the endoplasmic reticulum (ER) [29], [30], one such step may be export of *de novo* synthesized adiponectin from the ER due to stress imposed on ER by fatty acids [31], [32], [33], [34]. In fact, it has been recently demonstrated that ER stress may play a key role in obesity-induced suppression of adiponectin secretion [35]. Thus, we compared the effect of palmitate and the ER stress-inducing antibiotic, tunicamycin, on adiponectin secretion. As is shown in Fig. S2, both tunicamycin and palmitate decrease adiponectin secretion from 3T3-L1 adipocytes. The effect of tunicamycin is quite expected, as ER stress should, by definition, inhibit biosynthesis of secretory proteins. It becomes apparent after 8 h of incubation, likely because it takes several hours for newly synthesized adiponectin to travel through the secretory pathway and to be released into the media. On the contrary, palmitate inhibits adiponectin secretion already after 3 h of incubation, much prior to the ER stress-mediated inhibition of adiponectin secretion. The acute effect of palmitate suggests that it may promote degradation of *de novo* synthesized adiponectin in the adipocyte. In agreement with this hypothesis, we have found that incubation of cultured adipocytes with palmitate increases lysosomal staining and decreases the abundance of adiponectin in cells (Fig. 7A, B). In addition, the lysosomal inhibitor chloroquine alleviates the inhibitory effect of palmitate on adiponectin secretion (Fig. 7C). Thus, palmitate may stimulate lysosomal degradation of newly synthesized adiponectin.

**Figure 8:**
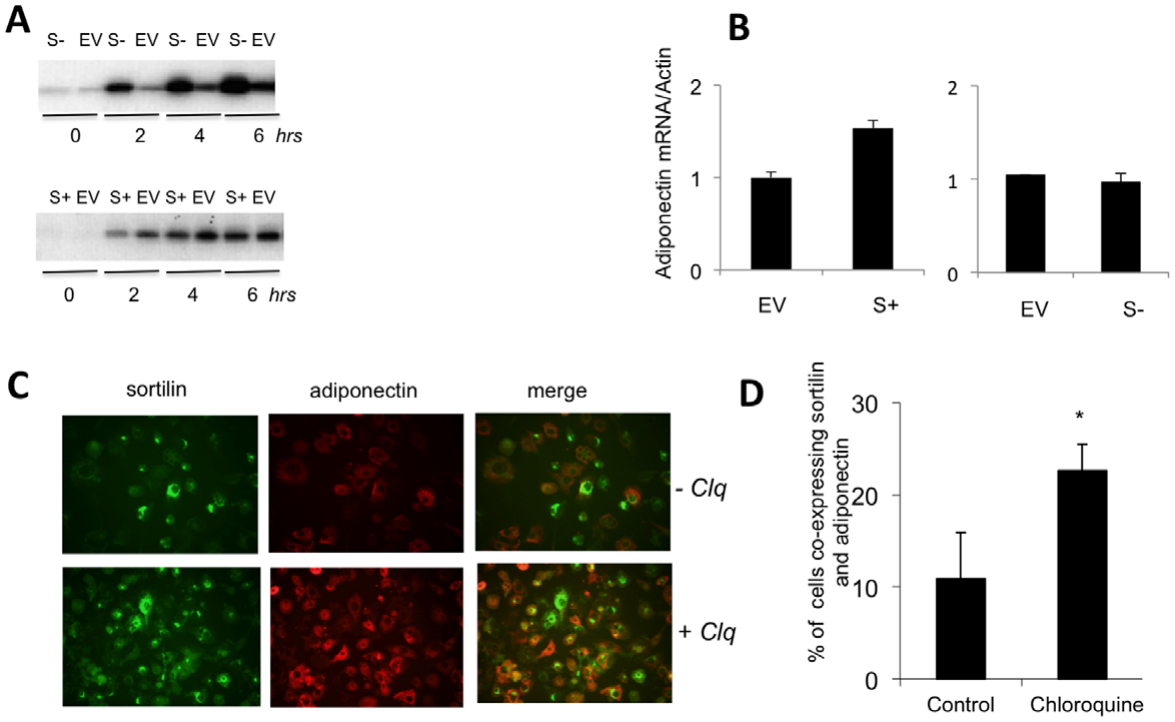
Sortilin is involved in lysosomal degradation of adiponectin. (A) Differentiated S-, S+ and control (empty vector-infected, EV) adipocytes were cultured in FBS replacement media for indicated periods of time, and secreted adiponectin was analyzed by Western blotting. (B) Adiponectin mRNA was analyzed in S-, S+ and control (empty vector-infected, EV) adipocytes by qPCR and normalized by actin. (C) S+ cells were treated and not treated with 20 µM Chloroquine for 4 hours and incubated with the mixture of primary monoclonal antibody against adiponectin and polyclonal antibody against myc overnight followed by incubation with donkey anti-mouse secondary antibody labeled with Cy3 (Jackson Immunoresearch, ME) and donkey anti-rabbit secondary antibody labeled with Alexa Flour488 (BD Biosciences, San Jose, CA) for 1 hr. (D) The number of cells that co-express sortilin and adiponectin with and without Chloroquine treatment was quantified and expressed as mean ± S.D. * p<0.05.

Mechanistically, lysosomal targeting of various proteins is mediated by a multi-ligand receptor, sortilin [36], [37], [38], [39], [40]. In order to determine whether or not sortilin is involved in lysosomal degradation of adiponectin, we used 3T3-L1 adipocytes stably over-expressing sortilin tagged with myc/His epitopes at the C-terminus (S+ cells) and 3T3-L1 adipocytes where sortilin expression had been knocked down with the help of shRNA (S- cells) [41]. We found that S+ adipocytes secrete less adiponectin than control empty vector-infected cells. Correspondingly, S- adipocytes secrete much more adiponectin that control cells (Fig. 8A). Apparently, these effects take place at a post-transcriptional level, as knock down of sortilin does not change the level of adiponectin mRNA determined by qPCR while over-expression of sortilin increases it (Fig. 8B). Also, there were no detectable changes in lipid accumulation and expression of multiple differentiation markers between S+ and S- adipocytes (not shown; see also [41]), suggesting that changes in adiponectin secretion are not explained by differences in cell differentiation.

As Western blotting “averages” adiponectin secretion from a large number of cells that may express different amounts of exogenous sortilin, we decided to continue this work by analyzing individual cells. For that, we performed double immunofluorescence staining of S+ adipocytes for sortilin and adiponectin. Using this approach, we found that cells with the highest levels of sortilin over-expression have virtually no adiponectin and *vice versa* (Fig. 8C). Quantification of these results demonstrates that only ca. 10% of cells over-expressing detectable amounts of exogenous sortilin contain adiponectin (Fig. 8D). Treatment of cells with chloroquine increased the number of S+ cells that co-express sortilin and adiponectin 2.5-fold (from 10 +/− 6% to 25 +/− 4%). These results are consistent with the idea that a significant fraction of intracellular adiponectin undergoes lysosomal degradation *via* a sortilin-mediated pathway.

## Discussion

Several research groups have reported that consumption of saturated fat suppresses production of the insulin-sensitizing adipokine adiponectin in white adipose tissue of humans and rodents (reviewed in [42], [43]. Furthermore, Fernandez-Real et al. demonstrated a negative correlation between palmitate and circulating adiponectin in human serum [44]. Thus, the inhibitory effect on the production of adiponectin may represent an important component of the diabetogenic action of saturated fatty acids.

In order to explore the molecular mechanism of this effect, we studied production of adiponectin by 3T3-L1 adipocytes in the presence and in the absence of palmitate. We found that palmitate rapidly inhibits the release of adiponectin from adipocytes. In order to search for an explanation of this effect, we found that palmitate inhibits transcription of the adiponectin gene. At the same time, expression levels of mRNAs for PPARγ and C/EBPα that control adiponectin transcription do not change (Fig. 1C). In addition, it was shown previously that palmitate did not decrease, but on the opposite, stimulated transcription of other adipokines, such as TNFα, IL6 [45], [46] and RBP4 (results not shown). Thus, the inhibitory effect of palmitate cannot be related to the general action of saturated fatty acids on transcription but is specific for transcription of adiponectin. This result is consistent with the previous findings showing that palmitate decreases adiponectin mRNA in adipocytes [47], [48]. It is well known, however, that isolation of primary adipocytes with the help of collagenase digestion carried out by Xi et al. [48] down-regulates multiple adipocyte genes in a non-specific fashion [49] rendering their results prone to alternative interpretations. The second study [47] demonstrated a decrease in adiponectin mRNA after 48 h incubation with palmitate which did not exclude potential indirect secondary effects of palmitate treatment. We studied the effect of palmitate after a relatively short 4 h incubation period which is likely to show a direct effect on adiponectin transcription. Still, our results suggest that palmitate *per se* is not likely to affect adiponectin gene expression. We favor the model according to which palmitate is rapidly metabolized in an ACSL1-dependent fashion into an as yet unidentified lipid product(s) that triggers phosphorylation of PPARγ on Ser273. Interestingly, knock down of ACSL1 increased phosphorylation levels of PKCθ and JNK – two protein kinases that are usually thought to mediate lipotoxic effects [27]. Therefore, increased adiponectin expression in ACSL1-ablated cells cannot be explained by suppression of these enzymes. Our results support the model of Choi et al. [19] according to which the effect of lipotoxicity on the adiponectin gene expression is mediated by CDK5.

We have also found that the amount of adiponectin produced by fat cells may be regulated at the level of protein degradation. This effect may not be specific for adiponectin as a large fraction of *de novo* synthesized secreted proteins undergoes lysosomal degradation in adipocytes. For example, up to 80% of LPL molecules newly synthesized in adipocytes entry the degradative pathway [50], [51], [52], [53]. In addition, up to 50% of newly synthesized leptin is also degraded instead of being secreted [54]. Importantly, degradation of LPL is mediated by sortilin [40]. More recently, it has been shown that “over-production” and sortilin-mediated degradation of secreted proteins is not unique to adipocytes. Thus, sortilin mediates lysosomal degradation of members of the TGF-β family [37] and brain-derived neurotrophic factor [36] in various mammalian cells.

It is yet hard to tell why the cell decides to operate in such a way; it is clear, however, that “futile cycles” of biosynthesis and degradation are often seen in metabolism. One possibility is that such mechanism may provide a faster response to rapid changes in metabolic conditions. According to this idea, the cell may prefer to re-route secreted proteins from degradation to secretion and *vice versa* rather than to induce/suppress protein expression that takes much longer time. Other factors, however, may also play a role. For example, it is feasible that the secretory capacity of the adipocyte is limited so that intracellular adipokines compete for being released into the extracellular space. In any way, it may be possible to take advantage of such situation in order to facilitate the release of adipokines of our choice and to inhibit secretion of unwanted proteins. Further experiments should uncover molecular details that are required to operate protein flows in adipocytes and other cell types.

## Materials and Methods

### Antibodies

In this study, we used monoclonal antibodies against adiponectin (Abcam, Cambridge, MA) and actin (Sigma Chemical Co., St. Louis, MO) and polyclonal antibodies against PPARγ, C/EBPα (Santa Cruz Biotechnology, Santa Cruz, CA), and myc epitope (Cell Signaling, Danvers, MA). Polyclonal phospho-specific antibody against pSer273 PPARγ was a kind gift of Dr. Spiegelman (Harvard Medical School).

### Cell culture

Wild type 3T3-L1 cells [55], 3T3-L1 cells infected with lentivirus carrying shRNA against ACSL1 and scrambled control [27], 3T3-L1 cells stably expressing sortilin tagged with myc/His epitopes at the C-terminus [41], and stable 3T3-L1 cell line with decreased expression of sortilin [41] were cultured, differentiated, and maintained as described previously [56]. Briefly, cells were cultured in Dulbecco's modified Eagle's medium (DMEM) supplemented with 10% bovine serum and 2 mmol/l L-glutamine, 100 units/ml penicillin, and 100 µg/ml streptomycin until confluency. Forty eight hours post confluency cells were differentiated using cocktail of insulin, dexamethasone, and isobutylmethylxanthine in DMEM supplemented with 10% fetal bovine serum (FBS), 2 mmol/l L-glutamine, 100 units/ml penicillin, and 100 µg/ml streptomycin. Mouse embryonic fibroblasts (MEFs) [18] and Swiss fibroblasts were grown in DMEM supplemented with 10% FBS, 2 mM L-glutamine, 100 units/ml penicillin, and 100 µg/ml streptomycin. The latter were stably transfected with the retroviral vector pBabe encoding either wild type PPARγ or S273A PPARγ. All procedures have been approved by the Institutional Biosafety Committee of Boston University Medical School (protocol 10-011, valid till 02/25/2013). In all secretion experiments, cells were incubated in 10% FBS replacement media (Lifeblood Medical Institute Adelphia, NJ) in DMEM. The stock solution of palmitate was prepared in two different ways with no apparent differences in the biological activity. First, palmitate was dissolved in 100% ethanol heated to 65°C to final concentration 10 mM. Alternatively, palmitate was dissolved in chloroform at 10 mM, and chloroform was evaporated under inert gas. The palmitate film was dissolved in potassium hydroxide at 1∶1.2 molar ratio of palmitate to KOH. This solution was mixed with 20% fatty acid free BSA in PBS at the molar ratio 3∶1.

### Western blot analysis

Proteins were run on SDS-polyacrylamide gels, transferred to Immobilon-P membranes (Millipore) in 25 mM Tris, 192 mM glycine. Following transfer, the membranes were blocked with 10% bovine serum albumin (BSA) in phosphate buffered saline (PBS) with 0.1% Tween 20 and probed with primary antibodies. Horseradish peroxidase-conjugated secondary antibodies (Pierce Biotechnology, IL) and an enhanced chemiluminescence substrate kit (Perkin-Elmer Life Sciences, MA) were used for detection of specific proteins. Blots were developed with the help of a Kodak Image Station 440CF (Eastman Kodak, Rochester, NY).

### RNA analysis

Total cellular RNA was prepared using TRIzol reagent (Ambion, Austin, TX) according to the manufacturer's instructions. Reverse transcription of 1μg of total RNA was done using random decamers (RETROscript kit; Ambion, Austin, TX), and mRNAs for adiponectin, PPARγ, C/EBPα, perilipin, FABP4, actin, GAPDH and 36B4 were determined by qPCR using MX4000 multiplex qPCR system (Stratagene, La Jolla, CA). All qPCR analyses were done in samples containing 2.5 µl of 1∶10 diluted cDNA, 22.5 µl of SYBR green master mix (Brilliant II SYBR Green qPCR master mix; Stratagene), and gene-specific primers (Operon, Huntsville, AL) in triplicate.

### Immunofluorescence

3T3-L1 adipocytes were grown and differentiated in 60-mm dishes. On day 5 of differentiation, cells were lifted up with 0.25% trypsin for 10 min at 37°C and reseeded to 4-well chamber slides. After growing on chamber slides for two more days, adipocytes were treated with 500 µM of palmitate for 4 hours and incubated with 100 nM of lysotracker (Invitrogen, Eugene, OR) for 30 minutes at 37°C. Cells were then washed 3 times with PBS and fixed with 4% paraformaldehyde in PBS for 30 minutes. Fixed cells were permeabilized with 0.2% Triton X-100 for 5 minutes and blocked with 5% donkey serum and 5% BSA for 1 hour. Cells were stained with primary monoclonal antibody against adiponectin overnight followed by secondary DyLight 488-coupled goat anti-mouse antibody (Jackson Immunoresearch, ME) at room temperature for 1 hour. Slides were mounted using Vectashield (Vector Laboratories, Burlingame, CA) and visualized with the help of Carl Zeiss immunofluorescent microscope Axio Observer Z1 (Germany).

### Statistics

Students paired two-tailed t-test was used to evaluate the statistical significance, and results were considered significant with the p value <0.05.

## Supporting Information

Figure S1 **Oleate decreases adiponectin expression and secretion.** (A) Differentiated 3T3-L1 adipocytes were incubated with and without oleate for 4 hours, and the presence of adiponectin in cell media was analyzed by Western blotting. Intracellular actin was used as a loading control. (B) Expression levels of mRNAs for adiponectin, PPARγ, and CEBPα in differentiated 3T3-L1 adipocytes incubated in the presence and in the absence of 500 µM of oleate for 4 hours. mRNA was analyzed by quantitative PCR and normalized by 36B4. Data were expressed as mean ± S.D. * p<0.05.
(TIFF)

Figure S2 **The effect of palmitate and tunicamycin on adiponectin secretion.** Differentiated 3T3-L1 adipocytes were incubated with 500 µM of palmitate or 2 µg/ml of tunicamycin for indicated periods of time, and the presence of adiponectin in cell media was analyzed by Western blotting. Intracellular actin was used as a loading control.
(TIFF)
