# Supplementary figures and images for: The Multi-Level Action of Fatty Acids on Adiponectin Production by Fat Cells
Source: PLoS One. 2011 Nov 29;6(11):e28146. doi: 10.1371/journal.pone.0028146 (PMC3226650; doi:10.1371/journal.pone.0028146)

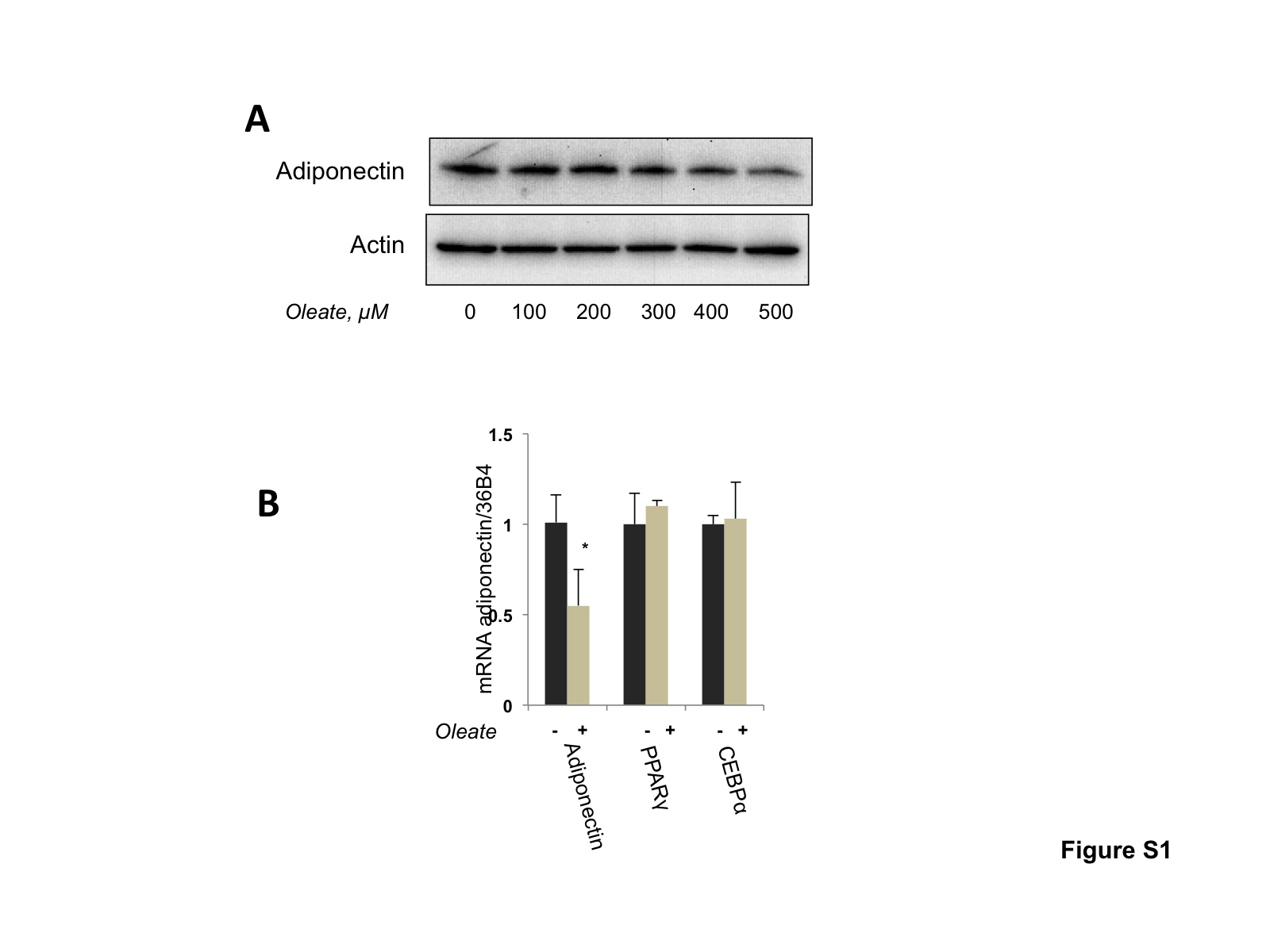

Supplement: Figure S1 — Oleate decreases adiponectin expression and secretion. (A) Differentiated 3T3-L1 adipocytes were incubated with and without oleate for 4 hours, and the presence of adiponectin in cell media was analyzed by Western blotting. Intracellular actin was used as a loading control. (B) Expression levels of mRNAs for adiponectin, PPARγ, and CEBPα in differentiated 3T3-L1 adipocytes incubated in the presence and in the absence of 500 µM of oleate for 4 hours. mRNA was analyzed by quantitative PCR and normalized by 36B4. Data were expressed as mean ± S.D. * p<0.05. (TIFF) [file pone.0028146.s001.tif]

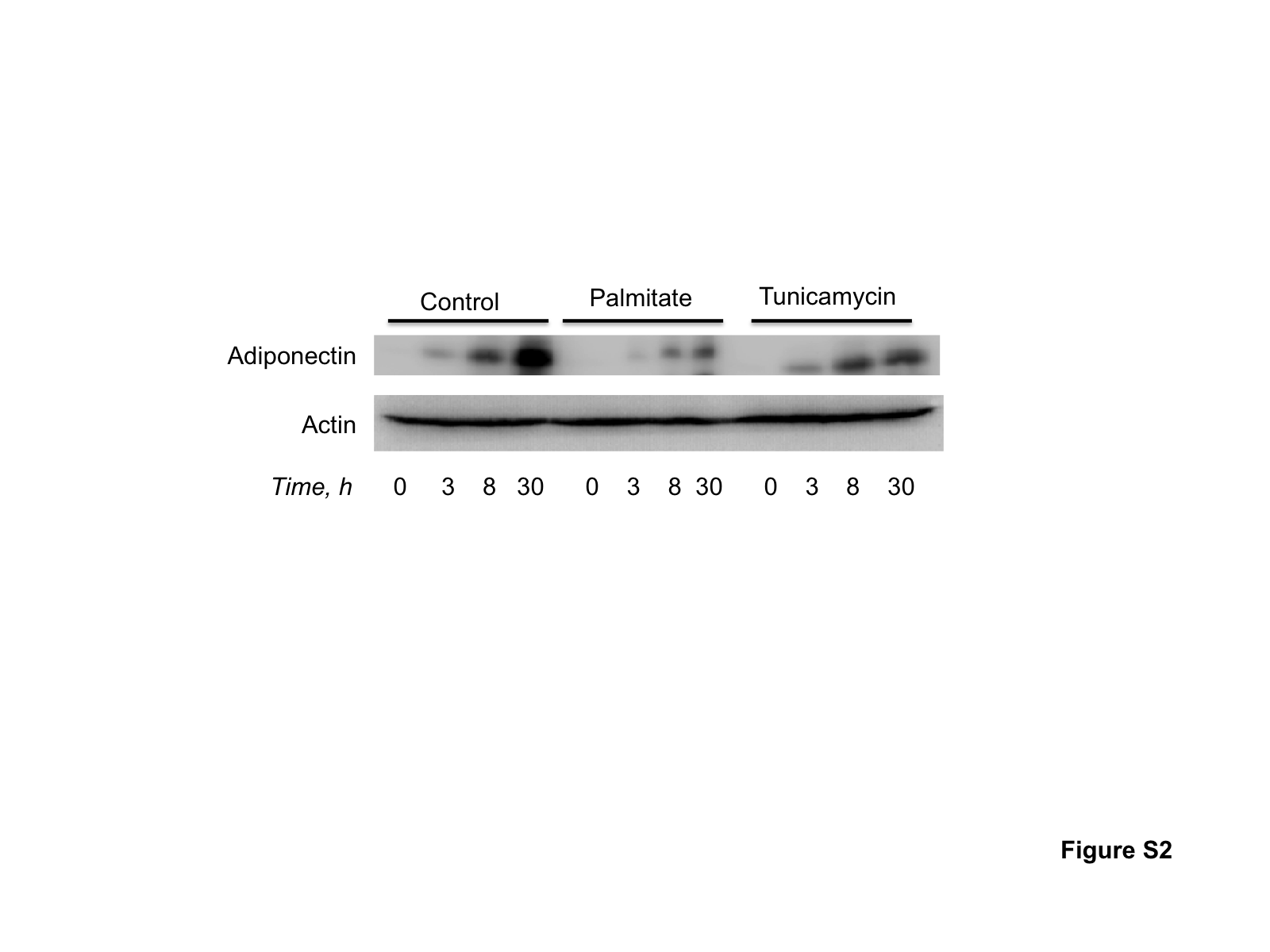

Supplement: Figure S2 — The effect of palmitate and tunicamycin on adiponectin secretion. Differentiated 3T3-L1 adipocytes were incubated with 500 µM of palmitate or 2 µg/ml of tunicamycin for indicated periods of time, and the presence of adiponectin in cell media was analyzed by Western blotting. Intracellular actin was used as a loading control. (TIFF) [file pone.0028146.s002.tif]
